# InAs/AlGaAs quantum dots for single-photon emission in a red spectral range

**DOI:** 10.1038/s41598-018-23687-7

**Published:** 2018-03-28

**Authors:** M. V. Rakhlin, K. G. Belyaev, G. V. Klimko, I. S. Mukhin, D. A. Kirilenko, T. V. Shubina, S. V. Ivanov, A. A. Toropov

**Affiliations:** 1 Ioffe Institute, 26 Politekhnicheskaya str., St. Petersburg, 194021 Russia; 2 St. Petersburg Academic University RAS, 8/3 Khlopina str., St. Petersburg, 194021 Russia; 3 ITMO University, 49 Kronversky pr., St. Petersburg, 197101 Russia

## Abstract

We report on comparative optical studies of InAs/Al_0.44_Ga_0.56_As quantum dots (QDs) grown by molecular beam epitaxy either with or without a thin GaAs interlayer inserted between the AlGaAs barrier and InAs QDs. Emission properties of individual QDs are investigated by micro-photoluminescence spectroscopy using 500-nm-size etched cylindric mesa structures. The single-photon statistics of the QDs of both types, emitting in the red spectral range between 636 and 750 nm, is confirmed by the measurements of the second-order correlation function. A negligibly small exciton fine structure splitting is detected in the majority of the QDs grown with the GaAs interlayer that implies the possibility of generating pairs of entangled photons with high entanglement fidelity.

## Introduction

The development of quantum cryptography and linear optic quantum computations^1–4^ relies on a nonclassical light source capable of emitting either a single photon or a pair of entangled single photons on demand at a defined frequency with high external quantum efficiency^5–7^. Self-organized single quantum dots (QDs) grown by epitaxial techniques are promising candidates for creation of the single-photon sources, because of their small emission linewidth, fast radiative decay time, high and stable quantum efficiency, and ability to be integrated with electronic devices^8^. Strain-induced QDs from a variety of different material systems, grown by the Stranski-Krastanov method, have been studied spectroscopically on a single QD level^9,10^, including InAs/GaAs, InP/(Al,Ga,In)P, CdSe/Zn(S)Se, CdTe/ZnTe, and more recently (In,Ga)N/(Al,Ga)N^11,12^, InAs/InAlGaAsP^13–15^, and InAs/InGaAs^16–18^. All together, these systems allow fabrication of the single-photon emitters in an extended spectral range from middle ultraviolet to the optical telecommunication C-band (1.55 *μ*m). Most of them, however, operate in a specific narrow spectral ranges so that any considerable variation of the emission wavelength requires strong modification or replacement of the expensive and complicated technological set-up.

The exception is the (In,Ga,Al)As material system, which allows design and fabrication of the single-photon emitters within a wide spectral range between ~900 nm (InGaAs/GaAs QDs) and ~1.55 *μ*m (InAs/InGaAs QDs). In this work, we expand the operational spectral range of the single-photon emitters fabricated in this system down to 636 nm, by means of InAs/AlGaAs Stranski-Krastanov QDs fabricated by molecular beam epitaxy (MBE)^19–22^. In these structures, the shift of the emission wavelength towards shorter wavelengths as compared with the most studied InAs/GaAs QDs arises from a combination of the larger barrier band gap and possible interdiffusion of Al into the QD material^20^. In particular, we focus on the practically important range 630–750 nm, which corresponds to the region of the highest sensitivity of modern single-photon avalanche diodes (SPAD) and is suitable for the development of protected lines of atmospheric and airspace optical communication^1^. Such single-photon sources can be also an indispensable part of quantum computers with an integrated optics architecture based on silica-on-silicon waveguide quantum circuits^23,24^, allowing room temperature operation due to replacement of currently exploited integrated cryogenic single-photon detectors based on superconducting nanowires^25,26^ by the integrated silicon-based SPADs. One should nevertheless emphasize that this spectral range does not apply for pure silicon photonic quantum computers^5,27^ due to enhanced optical losses in silicon chips at these wavelengths. Until recently, the single-photon emission in this range has been achieved using either InP based QDs^28^, or GaAs QDs grown by droplet epitaxy^29,30^. Disadvantage of the InP based heterostructures is a difficulty with growth of monolithic cavity structures, while the droplet-induced QD formation requires precise tuning of many growth parameters.

Generation of the polarization-entangled photons, realized with the use of a biexciton-exciton radiative cascade^31^, requires thorough control over exciton fine structure splitting (FSS), originating in the QDs with a zinc-blende structure from an asymmetric dot shape, strain, or piezoelectric effects^32^. Due to the respective reduction of the confinement symmetry (down to C_2*v*_ or lower), the anisotropic part of the electron-hole exchange interaction splits the exciton bright state into two eigenstates, which are linearly polarized along the crystallographic axes [110] and [1$\overline{1}$0]^33–35^. If the FSS is larger than the exciton homogeneous linewidth, it prevents the polarization entanglement of the emission^36,37^. Thus, the key condition of the generation of entangled photon pairs in a QD is the FSS reduction. The published data on the FSS values in the self-organized QDs, fabricated using the (In,Ga,Al)As material system, are sparse and contradictory. Finley *et al*.^20^ reported on observation of the FSS as large as ~1 meV in InAs/Al_0.6_Ga_0.4_As QDs emitting over the range between 1.55 and 1.64 eV (800 and 756 nm correspondingly). On the other hand, Gaisler *et al*.^22^ have recently observed a large number of Al_0.1_In_0.9_As/Al_0.24_Ga_0.76_As QDs (~30% of the total amount), emitting near 770 nm, with a record small FSS values below 10 *μ*eV. These data imply a complicated dependence of the exciton FSS in the InAs/AlGaAs QDs on precise details of the involved growth procedure.

In this paper, we present comparative optical studies of InAs/Al_0.44_Ga_0.56_As QDs grown by MBE either with or without an ultra thin GaAs interlayer inserted between the bottom AlGaAs barrier and InAs QDs. Emission properties of individual QDs are investigated by micro-photoluminescence (*μ*-PL) spectroscopy, which confirms the single-photon nature of the QDs emission in the red spectral range between 636 and 750 nm and demonstrates a pronounced effect of the GaAs interlayer formation, consisting in an essential decrease of the exciton FSS.

The rest of the paper is organized as follows. In the next section, we sequentially present and discuss the findings pertaining to general structural and optical characteristics of the InAs/AlGaAs QD structures, exciton FSS in the differently grown QDs, and statistical properties of the single-QD emission. Then, our conclusions are summarized. Finally, details of the measurement techniques are described in the section Methods.

## Results and Discussion

### Optical and structural characterization

**Figure 1:**
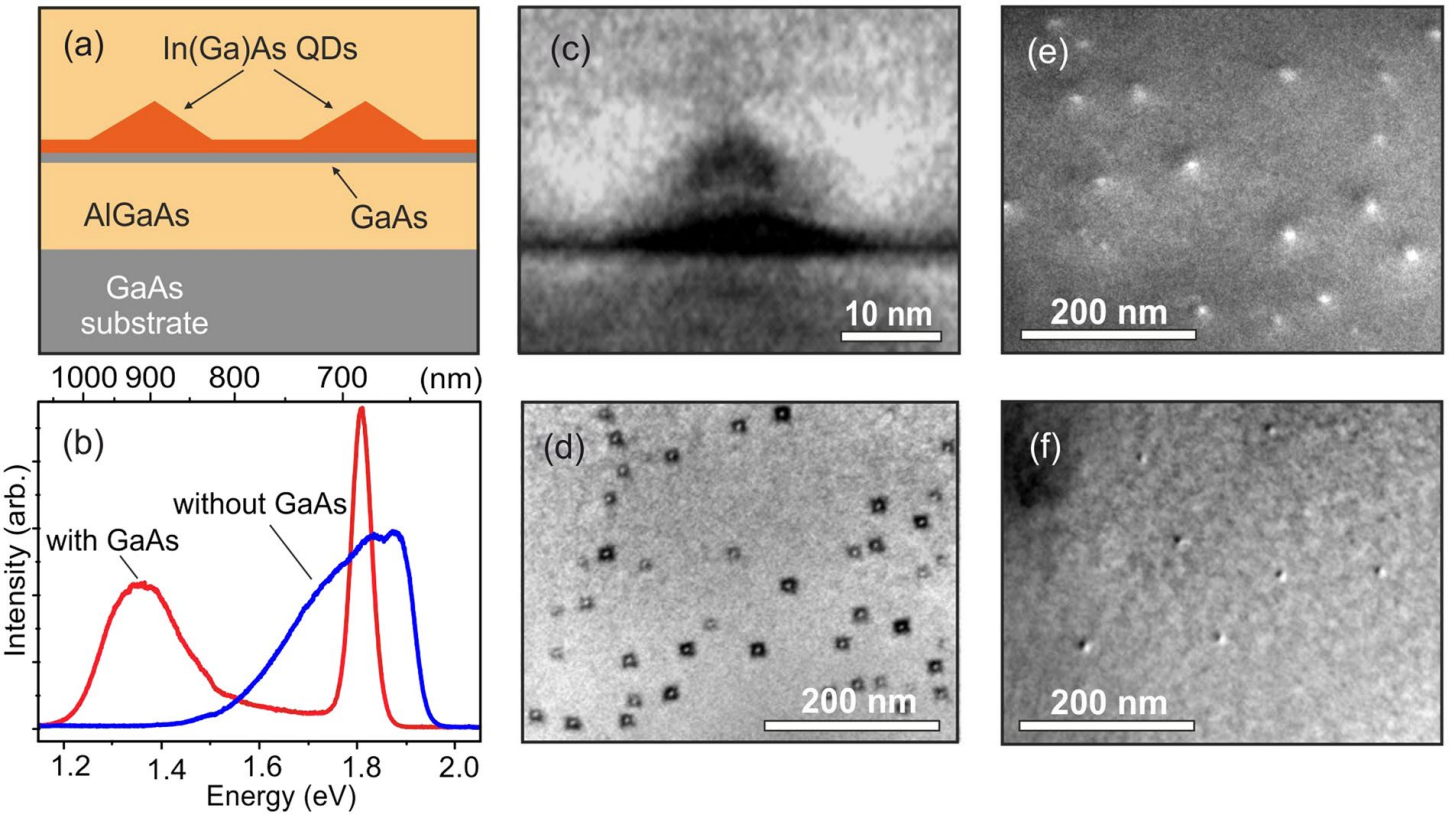
Schematic representation of investigated structures (**a**). Normalized spatially integrated PL spectra measured in samples A and B at 77 K (**b**). Cross-section darkfield (**c**) and plane-view bright-field (**d**) TEM images of sample A. Plane-view darkfield TEM images of sample A (**e**) and B (**f**).

The experiments were carried out using two series of samples fabricated by MBE, which contain self-assembled InAs QDs grown using the Stranski-Krastanow growth mode. In the samples of both kinds, the intended average thickness of the InAs deposition amounts to 1.7 monolayers (ML). The structures are grown on a GaAs (100) substrate capped with a 0.2-*μ*m thick GaAs buffer, which is followed by InAs QDs formed between 200-nm-thick bottom and 50-nm-thick top Al_0.44_Ga_0.56_As barrier layers at a growth temperature 510–520 °C and growth rate ~0.01 ML/s (Fig. 1(a)). In the samples of one series, QDs were grown with a predeposition of a 2-ML-thick GaAs interlayer, whereas in the samples of the other series, QDs were formed directly on the Al_0.44_Ga_0.56_As barrier layer. Further we discuss properties of two representative samples named as sample A (with the GaAs interlayer) and sample B (without the interlayer).

Surface density and geometrical parameters of QDs were estimated in samples A and B by means of transmission electron microscopy (TEM). Figure 1 shows the selective TEM images allowing estimation of the average QD surface density as ~10^10^ cm^−2^ in sample A and ~5 · 10^9^ cm^−2^ in sample B. Most of the QDs in sample A possess lateral sizes 10–20 nm and a height 3–4 nm. The QDs in this sample are square-based and show no indication for in-plane elongation (Fig. 1(d)). In sample B, accurate evaluation of the QD shape is hampered by a much weaker contrast of the TEM images, arising from the smaller In content in the alloyed QDs and/or smaller QD size along the growth direction.

The PL spectrum of sample A with the inserted GaAs interlayer demonstrates two separated peaks (Fig. 1(b)), which can be attributed to the emission of the QDs and a wetting layer (WL). The QD PL line is centered between 900 and 1000 nm that is similar to the emission of the InAs/GaAs QDs, while the higher energy tail of the line spreads up to the WL peak located near 690 nm. The emission line in sample B is markedly blue-shifted relative to that in sample A. This shift indicates smaller sizes of the formed QDs and, possibly, stronger incorporation of Al atoms into the QDs, induced by diffusion and/or segregation during growth. The QD emission line merges with the WL emission, spreading over the extremely wide energy range between ~1.4 and ~2 eV (890–620 nm).

**Figure 2:**
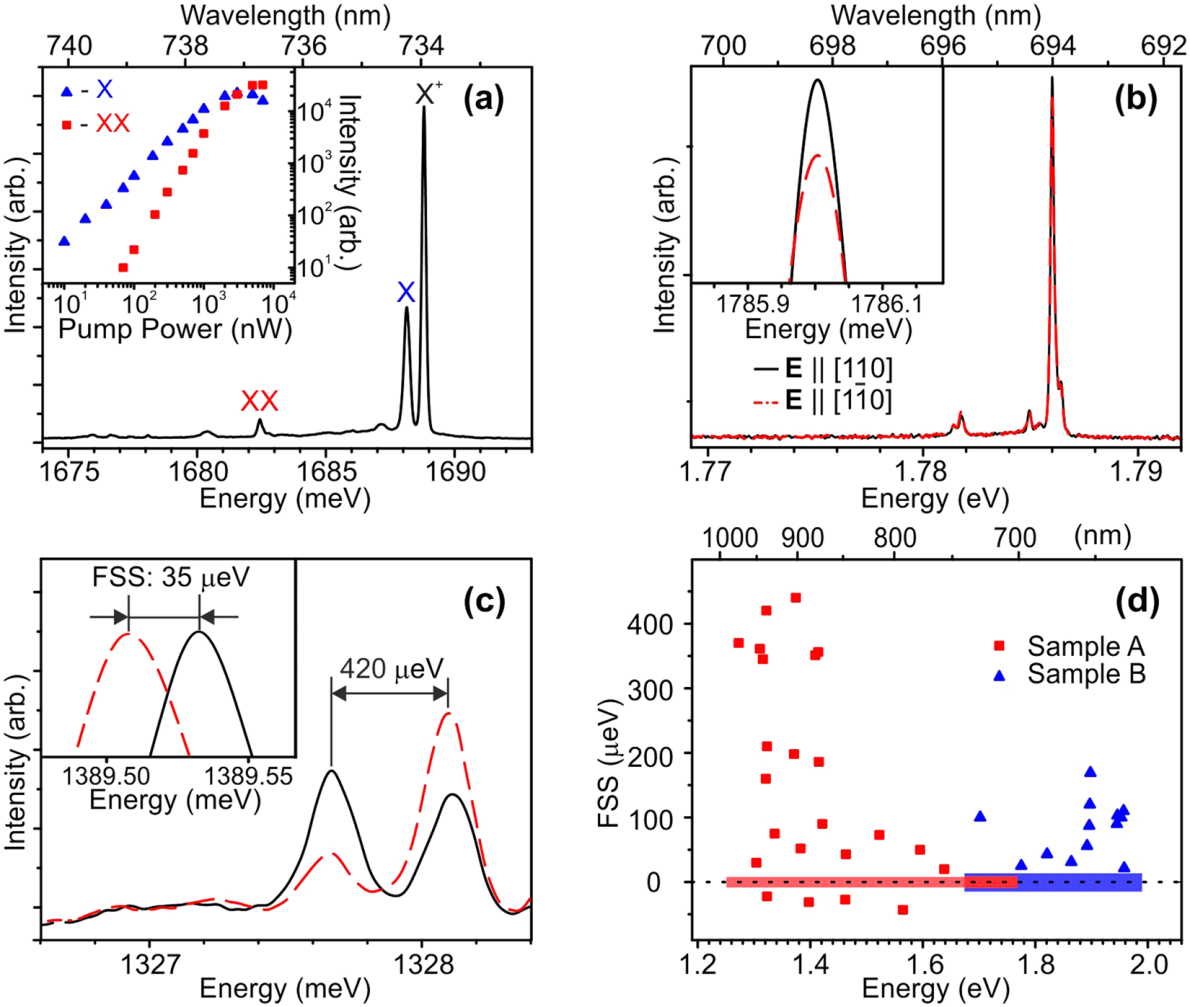
(**a**) *μ*-Pl spectra obtained in sample A, comprising emission lines of an exciton, a biexciton, and a positive trion. The insert shows a pump power dependence of the exciton and biexciton intensities. (**b**) Representative *μ*-PL spectra demonstrating nearly unsplit emission lines. The insert represents enlarged vertices of the lines. (**c**) An example of the emission lines with large positive FSS. The insert shows the vertices of the lines with negative FSS. Solid black and dash red lines in Figs (**b**,**c**) represent spectra for orthogonal linear polarizations corresponding to [110] and [1$\overline{1}$0] crystal directions of the QD sample. (**d**) Measured FSS as a function of the photon energy for samples A and B. The red and blue horizontal stripes correspond to unsplit emission lines with virtually zero FSS, detected in samples A and B, respectively.

The *μ*-PL spectra measured at 8 K reveal a number of relatively narrow lines (~100 *μ*eV), which can be assigned to the emission of neutral excitons (X) and electron-hole complexes: biexcitons (XX) and charged excitons (negatively (X^−^) and positively (X^+^) charged trions) in individual QDs. The origin of the particular lines can be identified by performing excitation- and polarization-dependent measurements (see Fig. 2)^38–40^. Thus, the exciton and biexciton emissions are distinguished by their characteristic excitation-power dependence, which is either linear (exciton) or quadratic (biexciton) (insert in Fig. 2(a)). In the experiments described further, the excitation power was kept low enough to ensure dominant excitation of neutral excitons rather than biexcitons. As for trions, either negative or positive, their emergence implies loading of a QD with an additional charge carrier (electron or hole), which is provided by doping or is injected optically due to the difference in the electron- and hole-diffusion lengths. In the InGaAs/GaAs QDs, the X^−^ emission line emerges 4.5–6.0 meV below X, whereas X^+^ line in most cases is slightly (~1 meV) blueshifted with respect to X^41^. The major signature of the singlet trion state is the complete lack of the emission line FSS. Nevertheless, in case of a small enough FSS value, unambiguous distinguishing between the weakly split excitonic lines and intrinsically unsplit trion lines can be problematic.

### Fine structure splitting

It was found that within the measurement accuracy many emission lines (~80%) in both samples are unsplit and nonpolarized. An example of such lines observed in sample A is shown in Fig. 2(b). In principle, they can be attributed either to the emission of excitons with negligibly small FSS, or to the emission of trions. The remnant part of QDs in this sample display different FSS in the range of values from nearly zero to the extremely large FSS of the order of 450 *μ*eV. Moreover, certain lines demonstrate small but distinct negative FSS, as shown in Fig. 2(c) demonstrating two doublet lines with different both signs and amplitudes of the FSS. Note that the FSS sign inversion was previously observed in the ensemble of InAs/GaAs QDs with small sizes^39^. All observed strongly split lines with certain linear polarization possess likely the strong asymmetry that reflects strong valence band mixing induced by the in-plain strain and shape anisotropy^42–45^. Such asymmetric QDs are definitely useless for the generation of the entangled photons. However, only few such lines were found in measured spectra, while the majority of the detected lines were only weakly split (if at all) that is a signature of the symmetric QDs.

The narrow emission lines in sample A emerge between 1.25 and 1.8 eV. All together, 118 randomly located lines were taken into account to estimate statistical distribution of FSS versus the emission photon energy. Among them, there are 95 lines with the FSS values less than the measurement accuracy (see Methods), which are considered as unsplit lines, and 23 split lines with measurable FSS values. The latter lines can be reliably attributed to the emission of neutral excitons. Filled squares in Fig. 2(d) represent the measured FSS of these excitonic lines as a function of the respective photon energy. The FSS is large and positive at small energies below 1.4 eV and rapidly decreases with the energy increase so that above ~1.5 eV the FSS values scatter around zero. By analogy with InAs/GaAs QDs^39^, this dependence indicates a rapid reduction of the FSS with a decrease in the QD size. The symmetric scattering of the data points around zero implies that at least a part of the “unsplit” lines, in fact, originate from the excitonic states with negligibly small FSS. Thus the smallest QDs emerging in sample A look very promising for both quantum cryptography and quantum computing applications, since they emit light near 750 nm, have small surface density, and possess negligibly small FSS.

In sample B, the statistical distribution of FSS values is essentially different (see filled triangles in Fig. 2(d)). There are 13 lines with measurable FSS among 84 lines, which were taken into account. These lines emerge between 1.7 and 2.0 eV and the respective average FSS values relatively weakly depend on the photon energy. The FSS sign is always positive and its amplitude scatters among different lines in the range of 10–180 *μ*eV. The nonsymmetric distribution of the FSS values around zero (absence of negative values) implies that most of the “unsplit” lines observed in this sample correspond to the truly nonpolarized emission of trions. Note that, although the singlet trion states are not suitable for the generation of entangled photon pairs, their application for ultrafast generation of single photons is preferable, as compared with neutral excitons, due to the absence of dark-state configurations^46^.

### Single-photon emission

**Figure 3:**
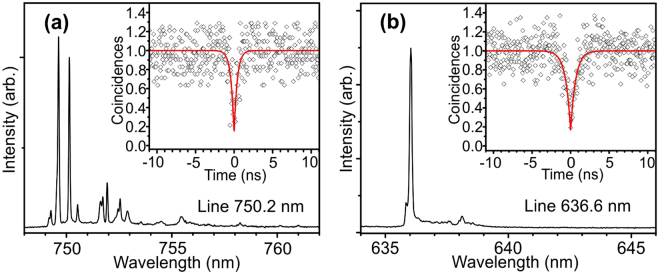
Normalized second order correlation function (*g*^(2)^) of single photon emission, measured at 8 K for excitonic lines in A (**a**) and B (**b**) samples. The obtained values of *g*^(2)^(0) are 0.17 (**a**) and 0.16 (**b**), respectively.

Figure 3 presents examples of autocorrelation function *g*^(2)^(*τ*) measured at T = 8 K within the narrow emission lines representative for samples A and B. The function is normalized according to the formula: *C*_*N*_(*t*) = *c*(*t*)/(*N*_1_*N*_2_*ωT*), where *c*(*t*) is raw coincidence number, *N*_1,2_ are single counters rates, *ω* is width of time bin, and *T* is a total acquisition time^47^. The shape of the functions is characteristic of excitonic lines and can be well fitted with the equation

1 $${g}^{(2)}(\tau )=a-{b}_{1}\,{{\rm{e}}}^{-|\tau |/{c}_{1}}.$$

For the measured correlation functions, the fitting gives the value of *g*^(2)^(0) < 0.2 that is a clear signature of the single-photon nature of the emission. The value at zero time does not reach zero as for a perfect single-photon emitter, most probably, because of presence of a background radiation, which contributes to the registered correlated counts. The achieved intensity of the single photon emission is about ~100 kHz being limited by the non-optimized extraction efficiency of photons in the simplified test mesa-structures. Further improvements in both purity and intensity of the emission imply the development of a cavity structure or a broadband waveguide nanoantenna with improved photons extraction efficiency^48^, as well as the realization of background-free resonance fluorescence^49^.

## Conclusion

To summarize, we have presented comparative studies of self-assembled InAs/Al_0.44_Ga_0.56_As QDs grown either with or without a GaAs interlayer. Both approaches allowed us to obtain QDs generating single-photon emission in the red visible spectral range. The QDs grown on top of the thin GaAs interlayer emit light in an extended range of 700–1000 nm. Especially useful for the development of the red single-photon sources are the QDs of smallest sizes, demonstrating small spectral density of the single-QD excitonic lines in the spectral range 700–750 nm and demonstrating negligibly small exciton FSS that is advantageous for the generation of entangled photon pairs. The structures grown without the interlayer are especially suitable for the single-photon generation at the shortest wavelengths down to 636 nm. These characteristics of single InAs/AlGaAs QDs make them promising for development of either cavity or photonic waveguide structures, intended for the generation of intense and pure quantum light in the red spectral range.

## Methods

### Measurements

TEM measurements were carried out using a Jeol JEM-2100F microscope. The emission of a limited number of individual QDs was detected by a confocal *μ*-PL set-up in cylindrical mesa-structures of 500 nm in diameter, which were fabricated by electron beam lithography and Ar^+^-ion etching for reducing the number of registered QDs.

*μ*-PL data were obtained at low temperature of 8 K. The samples were mounted in a He-flow cryostat with an Attocube XYZ piezo-driver inside, which allowed us to optimize and precisely maintain the positioning of a chosen mesa with respect to a laser spot during a long time (few hours). The luminescence was dispersed by a 0.5 m monochromator with a 1200/mm grating, providing direct spectral resolution ~70 *μ*eV. Nonresonant optical excitation by a *cw* laser line (405 nm) was used for the *μ*-PL measurements. The laser power density was below 4 W/cm^2^. The measurements were carried out in a backward geometry for orthogonal linear polarizations corresponding to [110] and [1$\overline{1}$0] crystal directions of the QD sample. Polarization control was achieved by means of a halfwave plate and a Glan-Taylor prism installed in the detection channel. In order to register the splitting between two linearly-polarized excitonic lines with the accuracy beyond the spectrometer resolution, the measured lines were fitted by Lorentzian curves and the maxima of the fitted contours were taken as the peak maxima. The limiting precision of thus measured FSS was estimated as ~20 *μ*eV.

Photon correlation measurements were performed in a Hanbury Brown-Twiss detection scheme exploiting two single-photon avalanche silicon diodes possessing the photon timing resolution of about 40 ps.

### Data availability statement

The datasets generated during and/or analyzed during the current study are available from the corresponding author on reasonable request.

## References

[1] Gisin N, Ribordy G, Tittel W, Zbinden H (2009). Quantum crypthography. Hum. Nat..

[2] Bouwmeester D, Ekert AK, Zeilinger A (2000). The physics of quantum information.

[3] Walls DF, Milburn GJ (2008). Quantum optics.

[4] Wang H (2017). High-efficiency multiphoton boson sampling. Nat. Photon..

[5] Rudolph T (2017). Why I am optimistic about the silicon-photonic route to quantum computing. Appl. Phys. Lett. Photon..

[6] Sangouard N (2007). Long-distance entanglement distribution with single-photon sources. Phys. Rev. A.

[7] Fedorov MV (2007). Anisotropically and high entanglement of biphoton states generated in spontaneous parametric down-conversion. Phys. Rev. Lett..

[8] Santori C, Fattal D, Yamamoto Y (2010). Single-photon device and applications.

[9] Bimberg D (2010). Semiconductor nanostructures.

[10] Mishler P (2009). Single semiconductor quantum dots.

[11] Holmes MJ, Choit K, Kako S, Arit M, Arakawa Y (2014). Room-temperature triggered single photon emission from a III-nitride site-controlled nanowire quantum dot. Nano Lett..

[12] Santori C (2005). Photon correlation studies of single GaN quantum dots. Appl. Phys. Lett..

[13] Kim JH, Cai T, Richardson CJK, Leavitt RP, Waks E (2016). Two-photon interference from a bright single-photon source at telecom wavelengths. Opt..

[14] Benyoucef M, Yacob M, Reithmaier JP, Kettler J, Michler P (2013). Telecom-wavelength (1.5 *μ*m) single-photon emission from InP-based quantum dots. Appl. Phys. Lett..

[15] Birowosuto D (2012). Fast Purcell-enhanced single photon source in 1,550-nm telecom band from a resonant quantum dot-cavity coupling. Sci. Rep..

[16] Chen Z-S (2017). Bright single-photon source at 1.3 *μ*m based on InAs bilayer quantum dot in micropillar. Nanoscale Res. Lett..

[17] Paul M (2017). Single-photon emission at 1.55 *μ*m from MOVPE-grown InAs quantum dots on InGaAs/GaAs metamorphic buffers. Appl. Phys. Lett..

[18] Al-Khuzheyri R (2016). Resonance fluorescence from a telecom-wavelength quantum dot. Appl. Phys. Lett..

[19] Polimeni A, Patane A, Henini M, Eaves L, Main PC (1999). Temperature dependence of optical properties of InAs/Al_*y*_Ga_1−*y*_As self-organized quantum dots. Phys. Rev. B.

[20] Finley JJ (2002). Fine structure for charged and neutral excitons in InAs-Al_0.6_Ga_0.4_As quantum dots. Phys. Rev. B.

[21] Grijseels SCM (2016). Radiative lifetimes and linewidth broadening of single InAs quantum dots in an Al_*x*_Ga_1−*x*_As. J. Lum..

[22] Gaisler AV (2017). AlInAs quantum dots. JETP Lett..

[23] Politi A, Cryan M, Rarity JG, Yu S, O’Brien JL (2008). Silica-on-silicon waveguide quantum circuits. Sci..

[24] Lee H, Chen T, Li J, Painter O, Vahala KJ (2012). Ultra-low-loss optical delay line on a silicon chip. Nat. Commun..

[25] Marsili F (2013). Detecting single infrared photons with 93% system efficiency. Nat. Photon..

[26] Akhlaghi MK, Schelew E, Young JF (2015). Waveguide integrated superconducting single-photon detectors implemented as near-perfect absorbers of coherent radiation. Nat. Commun..

[27] Suzuki K (2015). Ultra-high-extinction-ratio 2 × 2 silicon optical switch with variable splitter. Opt. Express.

[28] Ugur A (2012). Single-photon emitters based on epitaxial isolated InP/InGaP quantum dots. Appl. Phys. Lett..

[29] Mano T (2010). Self-assembly of symmetric GaAs quantum dots on (111)A substrates: suppression of fine-structure splitting. Appl. Phys. Express.

[30] Keil R (2017). Solid-state ensemble of highly entangled photon sources at rubidium atomic transitions. Nat. Commun..

[31] Benson O, Santori C, Pelton M, Yamamoto Y (2000). Regulated and entangled photons from a single quantum dot. Phys. Rev. Lett..

[32] Stier O, Grundmann M, Bimberg D (1999). Electronic and optical properties of strained quantum dots modeled by 8-band k · p theory. Phys. Rev. B.

[33] Gammon D, Snow ES, Shanabrook BV, Katzer DS, Park D (1996). Fine structure splitting in the optical spectra of single GaAs quantum dots. Phys. Rev. Lett..

[34] Bayer M (2002). Fine structure of neutral and charged excitons in self-assembled In(Ga)As/(Al)GaAs quantum dots. Phys. Rev. B.

[35] Goupalov SV, Ivchenko EL, Kavokin AV (1998). Anisotropic exchange splitting of excitonic levels in small quantum systems. Superlat. Microstruct..

[36] Santori C, Fattal D, Pelton M, Solomon GS, Yamamoto Y (2002). Polarization-correlated photon pairs from a single quantum dot. Phys. Rev. B.

[37] Stevenson RM (2006). A semiconductor source of triggered entangled photon pairs. Nat..

[38] Thompson RM (2001). Single-photon emission from exciton complexes in individual quantum dots. Phys. Rev. B.

[39] Seguin R (2005). Size-dependent fine-structure splitting in self-organized InAs/GaAs quantum dots. Phys. Rev. Lett..

[40] Rodt S, Schliwa A, Potschke K, Guffarth F, Bimberg D (2005). Correlation of structural and few-particle properties of self-organized InAs/GaAs quantum dots. Phys. Rev. B.

[41] Dalgarno PA (2008). Coulomb interactions in single charged self-assembled quantum dots: Radiative lifetime and recombination energy. Phys. Rev. B.

[42] Koudinov AV, Akimov IA, Kusrayev YG, Henneberger F (2004). Optical and magnetic anisotropies of the hole states in Stranski-Krastanov quantum dots. Phys. Rev. B.

[43] Belhadj T (2008). Impact of heavy hole-light hole coupling on optical selection rules in GaAs quantum dots. Appl. Phys. Lett..

[44] Tonin C (2012). Polarization properties of excitonic qubits in single self-assembled quantum dots. Phys. Rev. B.

[45] Harbord E (2013). Enhancement of valence band mixing in individual InAs/GaAs quantum dots by rapid thermal annealing. Jap. J. Appl. Phys..

[46] Strauf S (2007). High-frequency single-photon source with polarization control. Nat. Photon..

[47] Brouri R, Beveratos A, Poizat J-P, Grangier P (2000). Photon antibunching in the fluorescence of individual color centers in diamond. Opt. Lett..

[48] Cloudon J (2010). A highly efficient single-photon source based on a quantum dot in a photonic nanowire. Nat. Photon..

[49] Ding X (2016). On-demand single photons with high extraction efficiency and near-unity indistinguishability from a resonantly driven quantum dot in a micropillar. Phys. Rev. Lett..

